# Chitosan-Collagen 3D Matrix Mimics Trabecular Bone and Regulates RANKL-Mediated Paracrine Cues of Differentiated Osteoblast and Mesenchymal Stem Cells for Bone Marrow Macrophage-Derived Osteoclastogenesis

**DOI:** 10.3390/biom9050173

**Published:** 2019-05-05

**Authors:** Jeevithan Elango, Kandasamy Saravanakumar, Saeed Ur Rahman, Yves Henrotin, Joe M. Regenstein, Wenhui Wu, Bin Bao

**Affiliations:** 1 Department of Marine Bio-Pharmacology, College of Food Science and Technology, Shanghai Ocean University, Shanghai 201306, China; bbao@shou.edu.cn; 2 Bone Biology and Disease Unit, St. Vincent’s Institute of Medical Research, Melbourne, VIC 3065, Australia; 3 Department of Medical Biotechnology, College of Biomedical Sciences, Kangwon National University, Chuncheon, Gangwon 24341, Korea; saravana732@gmail.com; 4 Interdisciplinary Research Centre in Biomedical Materials (IRCBM), COMSATS University Islamabad, Lahore Campus, Islamabad 45550, Pakistan; saeedbio80@gmail.com; 5 Bone and Cartilage Research Unit, Arthropôle Liège, University of Liège, CHU Sart-Tilman, 4000 Liège, Belgium; yhenrotin@ulg.ac.be; 6 Department of Food Science, Cornell University, Ithaca, NY 14853-7201, USA; jmr9@cornell.edu

**Keywords:** chitosan-composite 3D matrix, rheological properties, biomechanical properties, mesenchymal stem cells, osteoclast, ovariectomized mice, RANKL, Runx2, bone homeostasis

## Abstract

Recent studies have identified the regulatory mechanism of collagen in bone ossification and resorption. Due to its excellent bio-mimicry property, collagen is used for the treatment of several bone and joint disease such as arthritis, osteoporosis, and osteopenia. In bone, the biological action of collagen is highly influenced by the interactions of other bone materials such as glycosaminoglycan and minerals. In view of the above perceptions, collagen was crosslinked with chitosan, hydroxyapatite (H), and chondroitin sulfate (Cs), to produce a natural bone-like 3D structure and to evaluate its effect on bone homeostasis using bone marrow mesenchymal stem cells, osteoblast, and bone marrow macrophages. The XRD and micro-CT data confirmed the arrangement of H crystallites in the chitosan-collagen-H-Cs (CCHCs) three-dimensional (3D)-matrix and the three-dimensional structure of the matrix. The stimulatory osteoblastogenic and exploitive osteoclastogenic activity of 3D-matrices were identified using differentiated osteoblasts and osteoclasts, respectively. Besides, osteogenic progenitor’s paracrine cues for osteoclastogenesis showed that the differentiated osteoblast secreted higher levels of RANKL to support osteoclastogenesis, and the effect was downregulated by the CCHCs 3D-matrix. From that, it was hypothesized that the morphology of the CCHCs 3D-matrix resembles trabecular bone, which enhances bone growth, limits bone resorption, and could be a novel biomaterial for bone tissue engineering.

## 1. Introduction

Over the past few years, there has been increasing demand for 3D matrices using different types of biopolymers to construct better biomaterials that provide the actual microenvironment for cellular response and signaling [1]. Several biopolymers from natural and synthetic materials have been used to construct 3D matrices for bone tissue engineering applications [2]. Among them, chitosan and collagen have established better bio-mimicry properties and maintain cell homeostasis [3,4]. In addition, chitosan has prospective benefits for cartilage tissue regeneration due to their resemblance to glycosaminoglycans, a component of cartilage matrix [5]. In general, the polysaccharide chitosan is produced from chitin by deacetylation, which influences the physicochemical, functional, and biological activity of chitosan [6]. 

The novel biomaterials chitosan and collagen are isolated from marine sources that have been widely used for many applications in the food and biomedical industries. They were used in the form of films, gels, 3D matrices or scaffolds, and microspheres in tissue engineering. It is recommended to culture cells in a three-dimensional (3D) matrix, since it gives the actual microenvironment, a similar structure to the biological architecture. However, understanding cell–matrix interaction is pivotal for the development of suitable 3D matrices for bone tissue regeneration. The empirical evidence of two-dimensional (2D) culture systems in cell growth has been studied for several decades, which provides considerable knowledge to determine how cells proliferate, differentiate, and trigger signals for homeostasis [7]. However, 2D culture systems for cell culture are not representative of the real cellular (3D) environment found in the body [8]. Therefore, it is important to investigate the actual bone cellular signaling mechanism on the 3D matrix to mimic the physiological 3D environment. In general, bone remodeling is a spontaneous mechanism that occurs in the normal biological processes to maintain bone flexibility and rigidity. Many hormones and cytokines such as receptor activator of nuclear factor-kB ligands (RANKL), macrophage colony stimulating factor, interleukin (IL)-6, and oncostatin M have been implicated in the maturation of bone cells. Among these factors, RANKL has a major role in osteoclast formation and differentiation. It has been reported that osteoblasts produce more RANKL to support osteoclastogenesis [9]. In contrast, Nakashima et al. [10] specified that in vitro osteoclastogenesis was mainly supported by osteocytes, as a major source of RANKL, rather than bone marrow mesenchymal stem cells (BMMSC) and osteoblasts.

The rheological and osteogenic activities of chitosan 3D matrices have been widely studied by many researchers [2,11]. To improve the structural and biological activity, chitosan has been combined with several biopolymers such as collagen, gelatin, hydroxyapatite, and nanoparticles. However, the combined effect of chitosan with the important bone components such as collagen, hydroxyapatite, and chondroitin sulfate in bone biology remains unclear as a 3D matrix. Since the interaction of these biopolymers may directly influence the bone regeneration activity, the combined effect of a chitosan composite 3D matrix needs to be investigated for better understanding the biological role in bone homeostasis. Hydroxyapatite (H), a natural bone mineral, is a potential biomaterial used for orthopedic, dental, and maxillofacial applications due to its excellent biocompatibility, bioactivity, and osteo-conductivity [12,13,14,15]. Chondroitin sulfate (Cs) is an important sulfated glycosaminoglycan compound (proteoglycan) in bone that has been postulated to orchestrate joint function and bone regeneration [16]. 

In a previous study, a 3D matrix was developed with the combination of collagen and chitosan, which improved the mechanical properties and human osteoblast cell growth [17]. In view of the importance of the 3D architecture to mimic the in vivo microenvironment, in this study, a chitosan-collagen-based composite 3D matrix has been used to investigate osteoblast formation and the bone cell remodeling mechanism in vitro. Recent studies also documented the potential efficiency of the chitosan-collagen 3D matrix on bone tissue regeneration [18,19]. By considering the above conceptualization, the present experiment was designed to address the impact of chitosan with collagen, H, and Cs on the physicochemical, structural, thermal, and biomechanical properties of the 3D matrix and also their influence on bone formation and bone resorption to maintain bone homeostasis. 

## 2. Materials and Methods

### 2.1. Chitosan-Collagen-Based Bio-Mimic 3D Matrix Preparation

Chitosan with an 85% degree of deacetylation (molecular weight: 470 kDa, viscosity: 800 cP), chondroitin sulfate, gum Arabic, and hydroxyapatite were purchased from Sigma-Aldrich (Shanghai, China). Type I collagen was extracted as per a previous method from tilapia fish skin using 1% pepsin in 0.5 M acetic acid and purified using gel filtration chromatography [20]. Four different types of 3D matrices, i.e., chitosan-collagen (CC), chitosan-collagen-hydroxyapatite (CCH), chitosan-collagen-chondroitin sulfate (CCCs), and chitosan-collagen-hydroxyapatite-chondroitin sulfate (CCHCs), were prepared. The composition of each 3D matrix was as follows: CC 3D matrix, 1% collagen and 1% chitosan; CCH 3D matrix, 1% collagen, 1% chitosan, and 0.5% hydroxyapatite; CCCs 3D matrix, 1% collagen, 1% chitosan, and 0.25% chondroitin sulfate; and CCHCs 3D matrix, 1% collagen, 1% chitosan, 0.5% hydroxyapatite, and 0.25% chondroitin sulfate. Gum Arabic (0.1%) was used as a plasticizer due to its potential use in bone growth. The matrix-forming solution was prepared with the addition of the respective composite in 0.5 M acetic acid and stirred for 4 h at 4 °C with a magnetic stirrer (1000 rpm) (Model: H97-A, Shanghai Mei Yingpu Instrument Manufacturing Co., Ltd. Shanghai, China) to produce a foam. The foam was immediately injected into a plastic tube (2 cm in diameter and 5 cm long) and kept at −80 °C overnight. The tubes were then lyophilized in a freeze drier, and chitosan-collagen-based 3D (CB3D) matrices were characterized. All the CB3D matrices were sterilized with 70% ethanol overnight in a shaker and washed three times with PBS for 15 min each and incubated with their respective cell culture medium for 2 h twice in a shaker and then used to grow cells. Matrix conditioned medium (MCM) was obtained by incubating the matrix in cell culture medium at the ratio of 1 mL medium to 10 mg of the matrix for 48 h and centrifuged at 1250× *g* for 5 min. 

### 2.2. Chitosan-Collagen Based 3D Matrix Characterization

The compressive strength of the CB3D matrix was tested using a Universal Testing Machine (TA-XT Plus, Stable Micro Systems, Surrey, UK). Matrix porosity and water binding were determined using ethanol and phosphate-buffered saline (pH 7.4) as the suspension medium, respectively [17]. The shrinkage factor was derived from the difference between the areas obtained before and after immersion of the matrix in phosphate-buffered saline (pH 7.4) [17]. The phase and crystallinity of the matrix were evaluated using an XRD (ZEISS HZG4 high-resolution diffractometer, Carl Zeiss Jane Co., Jena, Germany) and Cu-Kα_1_ radiation at a current of 40 mA and an accelerating voltage of 40 kV. Spectra were recorded as 2θ from 5–70° at a scanning speed of 1°/min and a step size of 0.02°. The three-dimensional structure and quantitative measurements of the pore size of the matrix were determined using microcomputed tomography (µCT100 micro-CT system, Scanco Medical, Bruttisellen, Switzerland). Scans were done using medium-resolution settings with a source voltage of 70 E (kVp), and images were analyzed using software supplied from Scanco (Image Processing Language Version 5.6). The thermal stability of the matrix was assessed with a TG 209 F1 analyzer (Netzsch-Geratebau GmbH, Selb, Germany) scanning from 0–700 °C at a rate of 10 °C min^−1^ in a nitrogen atmosphere purged at 100 mL min^−1^.

### 2.3. Cell Culture

Mouse pre-osteoblastic (MC3T3-E1) and BMMSC (ZQ0465) cells were purchased from Sciencell Research Laboratory, Shanghai Zhong Qiao Xin Zhou Biotechnology Co., Ltd. (Shanghai, China) and were cultured at 37 °C in a CO_2_ incubator (Shanghai Hengyue Medical Instruments Co., Ltd., Shanghai, China). Primary osteocytes (pOC) were harvested as per a previous protocol [21] and were cultured in α-MEM supplemented with 10% fetal bovine serum (FBS) (Gibco, Shanghai, China) at 37 °C in a CO_2_ incubator. MC3T3-E1 cells were grown in standard tissue culture flasks using l-ascorbic acid-free α-MEM supplemented with 10% FBS (Sciencell, Cat. No. 0025), 1% l-glutamine, and 1% penicillin/streptomycin (P/S) solution (10,000 units/mL of penicillin and 10,000 μg/mL of streptomycin in a saline solution) (Sciencell, Cat. No. 0503). Bone marrow mesenchymal stem cells were cultured in mesenchymal stem cell culture medium (Sciencell, Cat. No. 7501) containing 10% FBS, mesenchymal stem cell growth supplement (1% MSCGS, Sciencell, Cat. No. 7552), and 1% P/S. The media was replaced every 3–4 days. Upon 80% confluence, the cells were trypsinized using 0.25% trypsin/EDTA solution (Sciencell), and the cell numbers were counted using an Invitrogen cell counter (Countess II Automated Cell Counter, ThermoFisher Scientific, Shanghai, China). 

In all cases, for osteogenic differentiation, MC3T3-E1 cells were grown in α-MEM containing 50 µg/mL l-ascorbic acid (Sigma-Aldrich, Shanghai, China), and BMMSC cells were grown in osteoblast medium (Sciencell, Cat. No. 4601) with the addition of osteoblast growth supplement (ObGS) (Sciencell, Cat. No. 4652) composed of 100 nM dexamethasone, 10 mM β-glycerolphosphate, and 0.05 mM 2-phosphate-ascorbic acid for 14 days.

### 2.4. Cell Differentiation

The sterilized CB3D matrices (CC, CCH, CCCs, and CCHCs) were placed on 24-well plates (Costar, Shanghai, China) and MC3T3-E1 and BMMSC cells at Passage 3 were seeded (5 × 10^4^ cells/matrix/well) on top of the matrices. Blanks consisted of cells grown in a 2D environment using 24-well plates (Costar). Cells were cultured in the corresponding osteogenic stimulatory culture medium as mentioned above. After differentiation, the cells were harvested from the 2D and 3D matrix using 0.25% trypsin/EDTA solution (Sciencell) and centrifuged at 1500 rpm for 5 min. The cell pellet was re-dissolved in 1 mL of culture medium and counted using the Invitrogen cell counter (ThermoFisher) at 0, 3, 7, and 14 days. 

### 2.5. Cellular Alkaline Phosphatase

The level of cellular alkaline phosphatase (ALP) was measured as per the previous protocol [21]. At each time point (0, 3, 7, and 14 days), cells were harvested with lysis buffer (10 mM Tris buffer, pH 7.4) and treated with ALP substrate and p-nitrophenyl phosphate (Sigma-Aldrich) and read at 410 nm using a plate reader (Bio-Rad Model 550, Shanghai, China). The same volume of sample was used to determine protein content using bicinchoninic acid (BCA) as per the manufacturer’s instructions (Sangon Biotech Co., Ltd. Shanghai, China). ALP activity was expressed as nmol/min/mg protein. The cellular calcium level of cell lysate was measured as per the manufacturer’s instructions (Abcam, Shanghai, China).

### 2.6. Cellular Mineral Levels

MC3T3-E1 and BMMSC cells were seeded at a density of 1 × 10^4^ cells/well in microtiter 48-well plates and treated with MCM for two weeks. The mineralization effect of the matrix on osteogenesis was confirmed by observing calcium and phosphate deposition in cultured cells using the Alizarin red staining and the von Kossa method [22]. The presence of apatite in cell matrices was confirmed using a PerkinElmer FT-IR Micrsocope Apotlight 400 (FTIR/NIR Spectrometer ATR). The cell layers collected in 50 mM ammonium bicarbonate were freeze dried, and KBr cell pellets were scanned from 4000–450 cm^−1^. The apatite content was determined based on the spectrally-integrated area of the phosphate and protein peaks (mineral-to-matrix ratio). 

### 2.7. Determination of Hydroxyproline and Collagen Content

To investigate the osteogenic stimulatory activity of the CB3D matrix, the level of collagen content synthesized by the BMMSC and MC3T3-E1 cells was measured using the hydroxyproline (Hyp) content. On Day 14, the CB3D matrices along with cells were removed from the culture plates and gently washed with PBS. They were hydrolyzed in 6 N HCl for 24 h at 100 °C, dried in a vacuum evaporator and solubilized in distilled water, and the Hyp content was determined [23]. The sample was mixed with chloramine-T and Ehrich’s reagent before reading at 560 nm. As postulated by Neuman and Logan [24], the total collagen content was calculated by multiplying the Hyp content with the factor of 7.46. The CB3D matrix without cells served as the blank, and the value was subtracted from the total collagen content of the test samples. 

### 2.8. Histological Staining

The cells grown on the chitosan-collagen-based 3D matrix were fixed with 0.4% paraformaldehyde (PFA) for 15 min, dehydrated with increasing ethanol concentrations, followed by paraffin embedding and cutting into 10-μm sections using a microtome. The sections were deparaffinized, rehydrated, and stained with hematoxylin and eosin. The cells grown in MCM were fixed with 0.4% PFA and stained with a solution of naphthol AS-MX phosphate and fast blue RR dissolved in distilled water for the detection of alkaline phosphatase. 

### 2.9. Immunocytochemistry and Western Blot

Total cellular proteins were isolated, quantified, and separated using 10% SDS-PAGE and transferred to PVDF nitrocellulose membranes (Invitrogen) using the iblot-2 dry blotting system (Invitrogen). Protein transferred membranes blocked with 5% BSA-PBST were incubated with primary antibodies such as anti-GAPDH (Cat No. ab181602), anti-Col_1_α_2_ (Cat No. ab208638), and anti-osteocalcin (Cat No. ab93876) (Abcam) overnight at 4 °C. Then, the membranes were incubated with secondary goat anti-rabbit IgG-HRP (Cat No ab6721, Abcam) for 1 h at 37 °C and exposed to the enhanced chemiluminescent reagent (Abcam). Images were captured with a Universal Hood II Gel Doc System (Bio-Rad, Rochester, NY, USA). For immunocytochemistry, cells were grown in Nunc™ Glass Bottom Dishes (Cat No. 150682, ThermoFisher Scientific) with MCM, fixed with 4% PFA for 15 min and permeabilized with 0.1% Triton X-100 for 15 min at room temperature (RT). Then, the cells were incubated with primary antibody (anti-Col_1_α_2_) overnight and DyLight 594-conjugated secondary antibody (goat anti-rabbit IgG H&L, Cat No. ab96885, Abcam). In another experimental setup, cells grown in MCM were fixed with paraformaldehyde (4% in PBS), followed by cell membrane permeabilization using Triton-X 100 for 15 min, respectively, and stained with FITC and DAPI. Images were captured using a confocal laser scanning microscope (Leica TCS SP8, Leica Microsystems CMS GmbH, Wetzlar, Germany). 

### 2.10. mRNA Expression

Bone marrow mesenchymal stem cells and MC3T3-E1 cells were seeded (250,000 cells/well) in 6-well microtiter plates (Costar) along with MCM. Controls consisted of uncoated (without samples) wells. The percentage of mRNA expression (GAPDH, COL I, OC, RUNX2, ALP, and RANKL) was determined at different time intervals. The primers used for the RT-PCR are shown in Supplementary Table S1.

### 2.11. Scanning Electron Microscope 

Bone marrow mesenchymal stem cells and MC3T3-E1 cells grown on the CB3D matrix were fixed with 4% PFA for 15 min, dehydrated using increasing ethanol concentrations, and sputter coated with gold (30 s, 20 mA) for analysis using a scanning electron microscope (SEM-S4800, Hitachi, Tokyo, Japan) with a 20-kV accelerating voltage.

### 2.12. The Effect of Chitosan-Collagen-Based 3D Matrix on Osteoclast Formation

#### 2.12.1. Osteoporotic Model

Female adult C57BL/6 11-week-old mice were purchased from Sino-British Sippr/BK Lab Animal Co., Ltd. (Shanghai, China), and fed with normal feed and given water ad libitum. After the adaptive period for 2 weeks (at 25 °C with a 12-h light/dark cycle), mice were divided into two groups: Ovariectomized (OVX) and sham. Mice were subjected to ovariectomies using a dorsal approach [25] and caged without any treatment for 14 weeks to develop osteoporosis. Animal experiment protocols and procedures were approved by the Shanghai Ocean University Institutional Animal Care and Use Committee (Permit No. 13-0012). All methods were used in accordance with the relevant guidelines and regulations of the Scientific and Ethical Care and Use of Laboratory Animals of Shanghai Ocean University.

#### 2.12.2. Isolation of Mouse Bone Marrow Macrophages

The bone marrow of the femur and tibia of wild and OVX-mice was flushed out with ice-cold PBS and was cultured in a T75 cell culture flask (Costar) (20 mL final volume) containing 50 ng/mL recombinant Macrophage colony-stimulating factor (mCSF) (R&D Industries, Minneapolis, MN, USA) in endotoxin-free RPMI-10% heat-inactivated fetal bovine serum. Then, mouse bone marrow macrophage (mBMM) (10^5^ cells/well/48 well plates) were treated with RANKL (50 ng/mL), mCSF (30 ng/mL), and rPTHr11 (50 nM/mL) as previously described [21,26,27].

#### 2.12.3. The Effect of Chitosan-Collagen-Based 3D Matrix on Paracrine Signals of Osteogenic Cells with Osteoclast Precursor

To understand the actual paracrine cues of osteogenic cells on mBMM derived-osteoclast formation, primary osteocytes (pOC), BMMSC (differentiated (diff) and undifferentiated (undiff)), and MC3T3-E1 (diff and undiff) were co-cultured with osteoclast precursor cells without any stimulators. BMMSC and MC3T3-E1 cells were differentiated as mentioned previously. MCM of CCHC was used due to its better osteogenic stimulatory activities. The diff and undiff cells (1 × 10^4^) seeded separately on 48-well plates were co-cultured with osteoclast precursor (mBMM) with or without MCM. After ten days, the osteoclast cells were Tartrate-resistant acid phosphatase (TRAP) stained and counted.

### 2.13. Statistics

The average mean values and standard error of the mean were calculated from three determinations, and statistical significance was determined using one-way analysis of variance (ANOVA). Individual differences between mean values were assessed using Duncan’s multiple range tests. The results were also statistically interpreted using SPSS 18.0 (SPSS 18.0 for Windows, SPSS Inc., Chicago, IL, USA) to determine the least significant differences (LSD) at a *p* < 0.05. Each experiment was repeated thrice.

## 3. Results

### 3.1. Characterization of Composite Chitosan-Collagen-Based 3D Matrices

**Figure 1:**
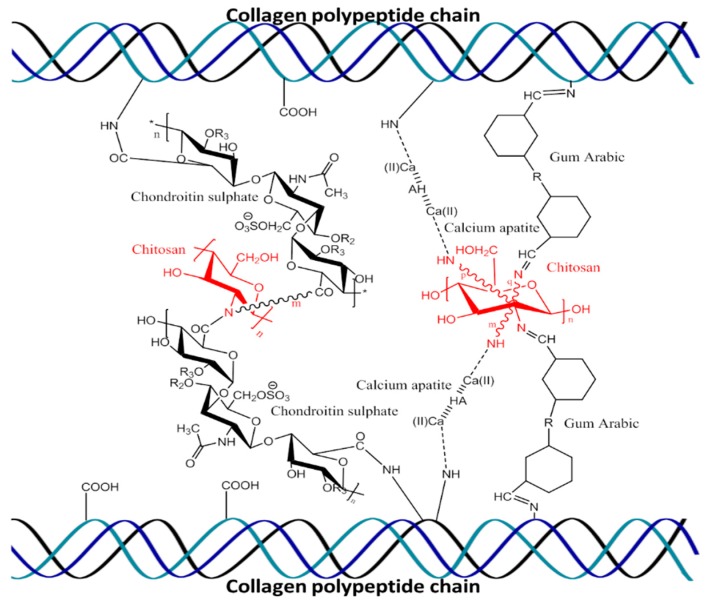
Schematic representation of molecular interactions of chitosan, gum Arabic, hydroxyapatite, and chondroitin sulfate with collagen polypeptides.

**Figure 2:**
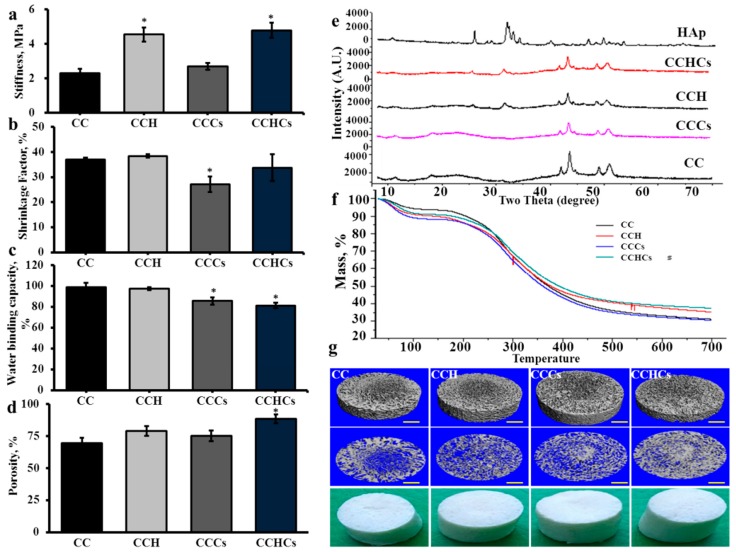
Rheological properties of chitosan-collagen-based bio-mimic three-dimensional (3D) matrices. Stiffness (**a**), shrinkage factor (**b**), water binding capacity (**c**), porosity (**d**), XRD spectra (**e**), TGA spectra (**f**), and micro-CT images (**g**) of chitosan-collagen-based bio-mimic 3D matrices. CC: chitosan-collagen 3D matrix, CCH: chitosan-collagen-hydroxyapatite 3D matrix, CCCs: chitosan-collagen-chondroitin sulfate 3D matrix and CCHCs: chitosan-collagen-hydroxyapatite-chondroitin sulfate 3D matrix. The experiments were done three times with similar results. * *p* < 0.05 vs. CC 3D matrix.

The possible interactions of materials—chitosan, collagen, H, Cs, and gum Arabic—in the 3D matrix are described in Figure 1. The addition of H significantly improved the compression strength and porosity of chitosan-collagen-hydroxyapatite (CCH) and chitosan-collagen-hydroxyapatite-chondroitin sulfate (CCHCs) 3D matrices (*p* < 0.05), while Cs had no interference in the chitosan-collagen-chondroitin sulfate (CCCs) 3D matrix (Figure 2a).

The shrinkage and water binding capacities were not affected significantly by the addition of H (Figure 2b–d); however, they were significantly decreased in CCCs compared to chitosan-collagen (CC) matrix. Two main XRD peaks were observed at 42° and 44°, corresponding to the characteristic peaks of collagen in all four CB3D matrices (Figure 2e). The 2θ angles of CB3D matrices’ XRD spectra were not similar due to the different molecular arrangement between matrices. However, diffraction peaks at 25.7°, 31.8°, 32.8°, and 33.8° in both CCH and CCHCs matrices were similar to the peaks observed in commercial hydroxyapatite, which indicated the presence of H crystallites, and those peaks were absent in C and CCCs matrices. From the TGA curves, 50% weight loss in C, CCH, CCCs, and CCHCs matrices was observed at 353, 393, 360, and 418 °C, respectively (Figure 2f). At a high temperature (700 °C), C, CCH, CCCs, and CCHCs matrices lost a total mass of about 31, 35, 30, and 41%, respectively. The micro-CT-based hierarchical structure of the CB3D matrix showed the mean pore size as 452, 312, 374, and 250 μm for C, CCH, CCCs, and CCHCs, respectively (Figure 2g). Compared to other scaffolds, the CCHCs matrix had a smaller pore size due to the presence of H and Cs.

### 3.2. Osteogenic Regulatory Effect of the Chitosan-Collagen-Based 3D Matrix

**Figure 3:**
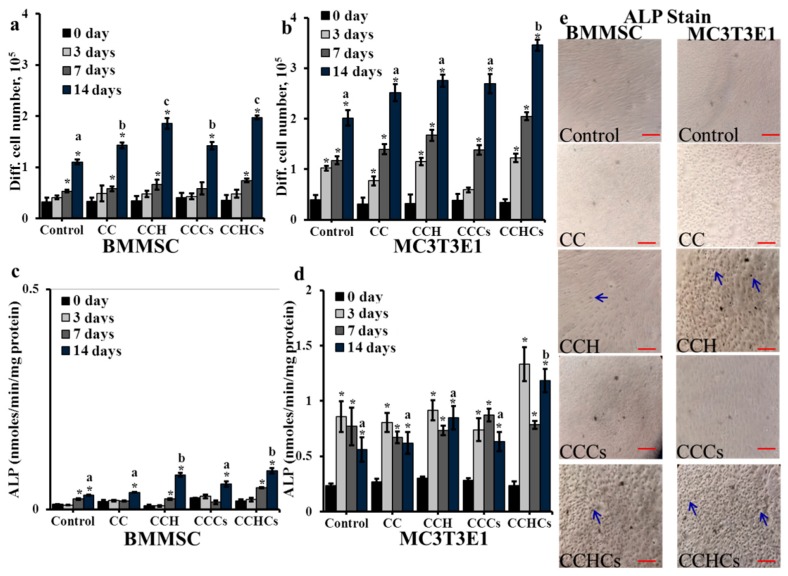
The effect of chitosan-collagen-based bio-mimic 3D matrices on bone cells’ differentiation (**a**,**b**), cellular alkaline phosphatase (ALP) (**c**,**d**) (Supplementary Figure S1 shows the level of ALP normalized with the cell number), and histological staining of alkaline phosphatase (**e**) using naphthol AS-MX phosphate and fast blue RR dye; cells were stained with naphthol AS-MX phosphate and fast blue RR after 14 days of culture and arrows show positively-stained cells. Scale bars: 40 μm. BMMSC: bone marrow-derived mesenchymal stem cells. CC: chitosan-collagen 3D matrix, CCH: chitosan-collagen-hydroxyapatite 3D matrix, CCCs: chitosan-collagen-chondroitin sulfate 3D matrix, and CCHCs: chitosan-collagen-hydroxyapatite-chondroitin sulfate 3D matrix. The experiments were done three (a–d) or two (e) times with similar results. * *p* < 0.05 vs. zero days; different letters indicate statistical significance among 3D matrices for 14 days.

In general, BMMSC and MC3T3-E1 cells’ growth was increased from 0–14 days in cultures (Figure 3a,b). As expected, the differentiation rate of both cells grown on the CCHCs matrix was accelerated significantly compared to cells grown on other matrices. On Day 14, a high cell differentiation rate was observed in the CCH and CCHCs matrices for BMMSC cells and in the CCHCs matrix for MC3T3-E1 cells (*p* < 0.05), which was further supported by the ALP level of bone cells (Figure 3c–e).

**Figure 4:**
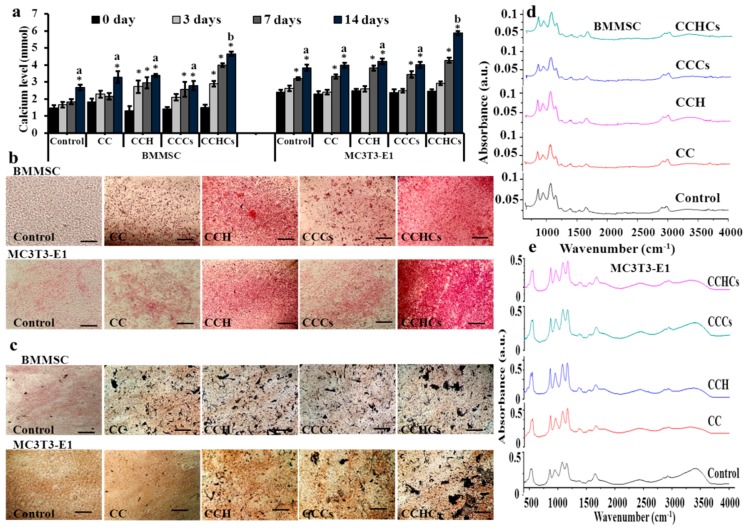
Cellular calcium deposition of bone cells cultured on chitosan-collagen based bio-mimic 3D matrices for 14 days. The cellular mineral level (**a**), Alizarin red (**b**), and von Kossa (**c**) staining of bone cells. Scale bars: 40 μm. The presence of apatite was confirmed using FTIR spectra (**d**,**e**); control-cells grown on a regular tissue culture plate. BMMSC: bone marrow-derived mesenchymal stem cells; MC3T3-E1, pre-osteoblast. CC: chitosan-collagen 3D matrix, CCH: chitosan-collagen-hydroxyapatite 3D matrix, CCCs: chitosan-collagen-chondroitin sulfate 3D matrix, and CCHCs: chitosan-collagen-hydroxyapatite-chondroitin sulfate 3D matrix. The experiments were done three (a) or two (b–e) times with similar results. * *p* < 0.05 vs. zero days; different letters indicate statistical significance among 3D matrices for 14 days.

Cellular calcium levels of differentiated BMMSC and MC3T3-E1 cells cultured on the CCHCs matrix increased gradually with culture duration (Figure 4a). Histological mineral staining of BMMSC and MC3T3-E1 culture using Alizarin red and silver nitrate showed the existence of high levels of nodular red precipitate and apatite black precipitate in the extracellular matrix (Figure 4b,c). The CCHCs matrix accelerated the amount of mineral deposition in MC3T3-E1 cell cultures compared to other matrices (C, CCH, and CCCs), which confirmed the osteoblastogenic potential of this matrix. The Fourier transform infrared (FTIR) spectroscopic assessment showed that the mineral to matrix ratio of osteoblastic lineage cells from BMMSC and differentiated MC3T3-E1 cells cultured on 2D (control culture), C, CCH, CCCs, and CCHCs were about 1.23, 1.57, 2.72, 2.68, and 3.5; and 1.76, 2.26, 3.18, 2.84, and 4.8, respectively (Figure 4d,e).

**Figure 5:**
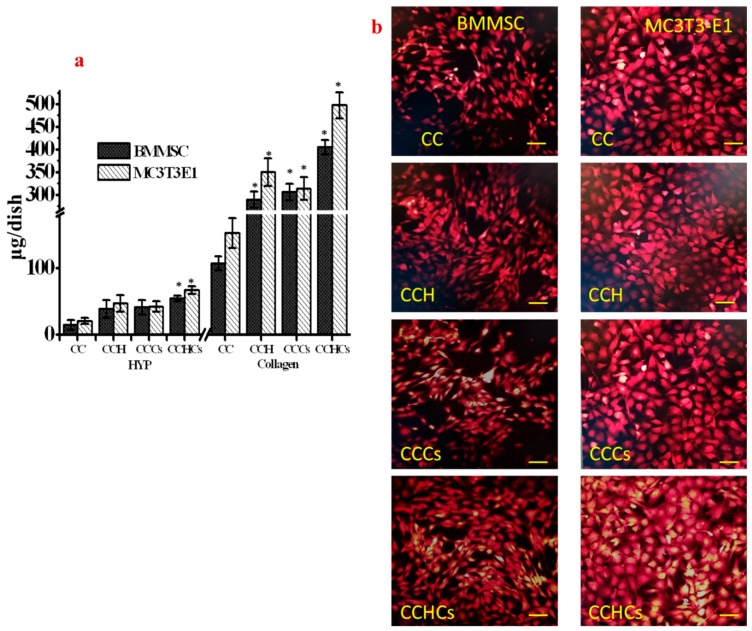
(**a**) The effect of chitosan-collagen-based bio-mimic 3D matrices on hydroxyproline and collagen synthesis in bone cells; * *p* < 0.05 vs. CC. (**b**) Immunocytochemistry evaluation of collagen I expression of bone cells cultured for 14 days. Scale bars: 100 μm. CC: chitosan-collagen 3D matrix, CCH: chitosan-collagen-hydroxyapatite 3D matrix, CCCs: chitosan-collagen-chondroitin sulfate 3D matrix, and CCHCs: chitosan-collagen-hydroxyapatite-chondroitin sulfate 3D matrix.

The CCHCs matrix triggered collagen synthesis compared to other matrices (Figure 5a,b). Indeed, cells grown on CCH and CCCs matrices produced more collagen than cells cultured in the C matrix, but there was no significant difference in the total collagen content between both matrices. MC3T3-E1 cells had synthesized high levels of collagen during the differentiation process compared to differentiation of BMMSC cells cultured on the CB3D matrix (Figure 5a). Cells cultured on CB3D matrices showed strong immunostaining with Col_1_α_2_ monoclonal antibodies, and the level of expression was higher in MC3T3-E1 cells than BMMSC cells (Figure 5b).

### 3.3. Cell Spatial Distribution on the Chitosan-Collagen-Based 3D Matrix

**Figure 6:**
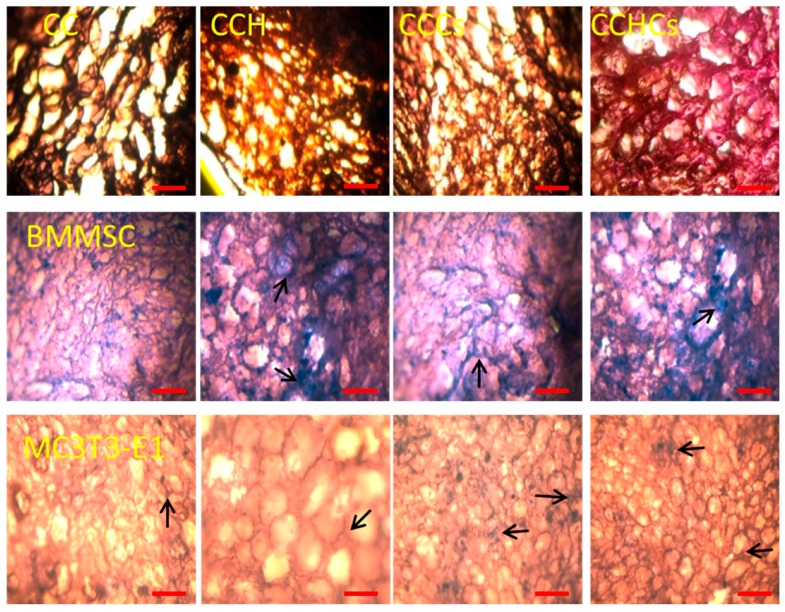
Hematoxylin-eosin staining of bone cells cultured on 3D matrices, 100× magnification. Cell nuclei are stained dark purple and 3D matrices pink. Scale bars: 200 µm. Arrows show stained cells. BMMSC: bone marrow-derived mesenchymal stem cells; MC3T3-E1, pre-osteoblast. CC: chitosan-collagen 3D matrix, CCH: chitosan-collagen-hydroxyapatite 3D matrix, CCCs: chitosan-collagen-chondroitin sulfate 3D matrix, and CCHCs: chitosan-collagen-hydroxyapatite-chondroitin sulfate 3D matrix.

The spatial distribution and interconnectivity of cells were affected by the different compositions of the CB3D matrices. Among CB3D matrices, the CCHCs matrix had a regular and uniform pore distribution, which showed good infiltration and adhesion of BMMSC and MC3T3-E1 cells (Figure 6). A cluster of differentiated MC3T3-E1 cells, stained a deep purple color, found on CCH, CCCs, and CCHCs matrices supported their osteogenic differentiation. Both osteoblastic lineage cells from BMMSC and differentiated MC3T3-E1 cells could easily adhere to the interior and surface of CB3D matrices.

### 3.4. Protein and mRNA Expression

**Figure 7:**
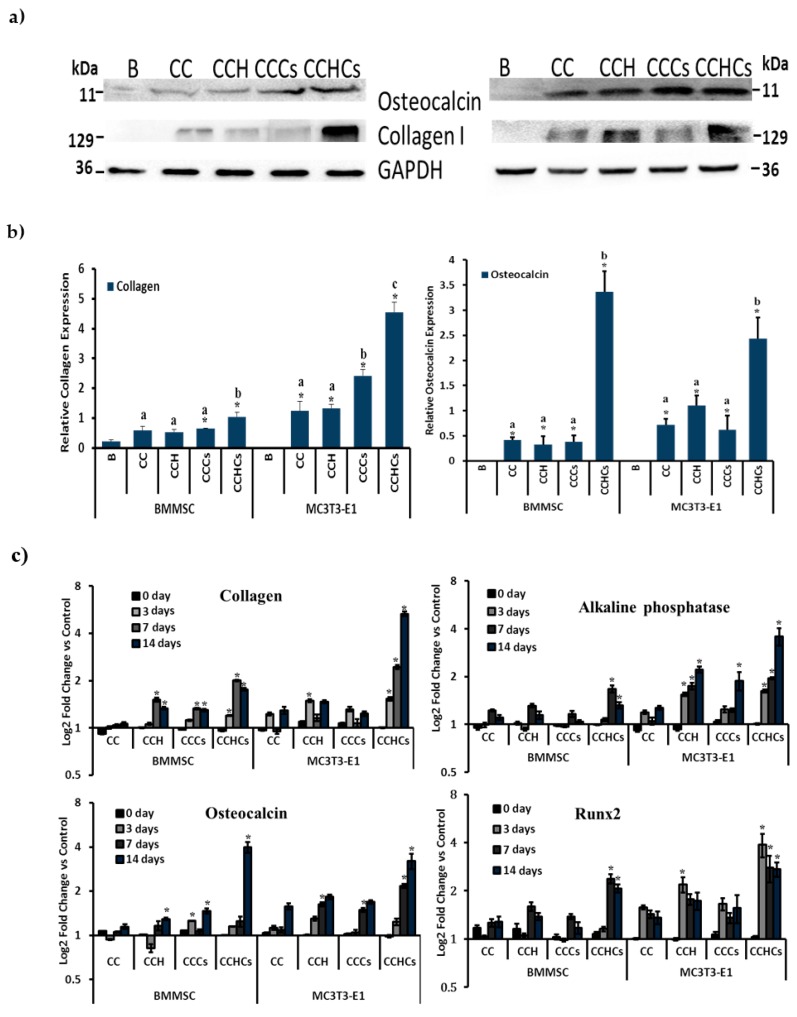
Protein (**a**,**b**) and mRNA expression (**c**) of bone cells. (**a**,**b**) Western blot analysis of collagen I and osteocalcin proteins expressed in bone cells. GAPDH was a loading control. (**c**) Osteogenic mRNA expression of differentiated bone cells cultured in 3D matrices. mRNA expression of bone cells was normalized with GAPDH. Blank (B)-cells cultured in a six-well culture plate. Expression of osteogenesis regulatory mRNA was normalized with GAPDH of bone cells cultured at different times (0, 3, 7, and 14 days). BMMSC: bone marrow-derived mesenchymal stem cells; MC3T3-E1, pre-osteoblast. CC: chitosan-collagen 3D matrix, CCH: chitosan-collagen-hydroxyapatite 3D matrix, CCCs: chitosan-collagen-chondroitin sulfate 3D matrix, and CCHCs: chitosan-collagen-hydroxyapatite-chondroitin sulfate 3D matrix. Data are from the experiment repeated thrice with similar results; * *p* < 0.05 vs. blank (**b**); * *p* < 0.05 vs. CC (**c**); different letters indicate statistical significance among 3D matrices.

To examine the effect of the CB3D matrix on the expression of osteogenic proteins in MC3T3-E1 and BMMSC cells, Western blotting antibodies directed against osteogenic proteins such as Col_1_α_2_ and osteocalcin were used. This approach showed that Col_1_α_2_ was increased in BMMSC-derived osteoblastic lineage cells and differentiated MC3T3-E1 cells cultured on the CCHCs 3D matrix compared to other matrices (Figure 7a). Cells cultured on CB3D matrices showed high Col_1_α_2_ protein expression, and the level of expression was higher in MC3T3-E1 cells than BMMSC cells (*p* < 0.05) (Figure 7b). To further analyze the mechanism leading to the differentiation of osteogenic cells on CB3D matrices, mRNA levels of the genes of interest were measured using RT-PCR in osteogenic cells cultured on the CB3D matrix (Figure 7c). Col_1_α_2_ and osteocalcin (OC) mRNA levels were significantly increased in differentiated MC3T3-E1 cells on the CCHCs matrix on Days 7 and 14. However, there were no significant changes observed in differentiated BMMSC cells on the CB3D matrices except on Day 14 in the CCHCs matrix.

Alkaline phosphatase and Runx2 mRNA levels were increased significantly between zero and 14 days in untreated BMMSC and MC3T3-E1 cells (*p* < 0.05). Except for the CCH and CCHCs matrix cultured BMMSC cells on Days 7 and 14, there was no significant difference in ALP mRNA levels between control and CB3D matrix cultured cells. On Day 14, ALP mRNA levels were increased in the CCH and CCHCs matrix-treated MC3T3-E1 cells (*p* < 0.05). On Day 14, Runx2 mRNA levels were significantly increased in BMMSC cells cultured on CCH and CCHCs compared to the control. In differentiated MC3T3-E1 cells, the level of Runx2 was increased significantly on Days 7 and 14 when cultured on CCH, CCCs, and CCHCs matrices compared to the control (*p* < 0.05).

### 3.5. Scanning Electron Microscope

**Figure 8:**
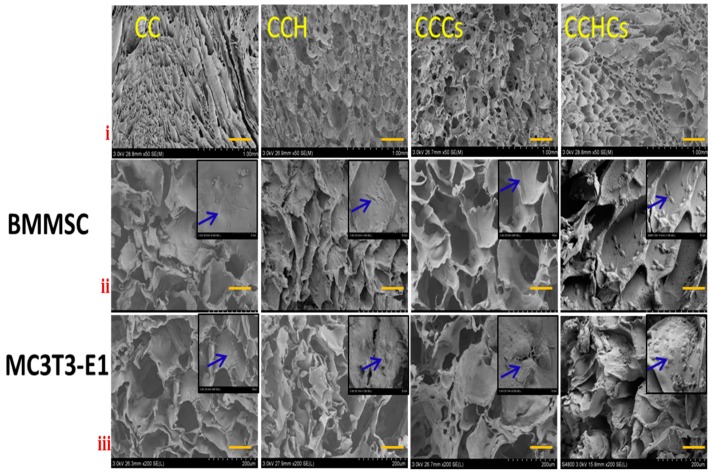
Scanning electron microscope (scale bars: i = 1.0 mm, ii and iii = 200 μm) images of bone cells cultured on 3D matrices for 14 days. The small inset in the SEM image is showing the expanded magnification at 50 μm. CC: chitosan-collagen 3D matrix, CCH: chitosan-collagen-hydroxyapatite 3D matrix, CCCs: chitosan-collagen-chondroitin sulfate 3D matrix, and CCHCs: chitosan-collagen-hydroxyapatite-chondroitin sulfate 3D matrix.

Scanning electron microscope images showed that all CB3D matrices had formed cavities with interconnections, but of different shapes and sizes. The decreased pore size of CCHCs matrices compared to other matrices was clearly seen in the SEM image (Figure 8), which confirmed the observations with the micro-CT. The pores of the C matrix were irregular in shape, size, and not uniformly distributed with a sheet-like structure. In the CCH, CCCs, and CCHCs matrices, the microstructure had more homogenous pores of uniform size, but with an absence of a large sheet-like structure. As shown in Figure 8, osteoblastic lineage cells from BMMSC and differentiated MC3T3-E1 cells attached and uniformly colonized almost all the surfaces of the CB3D matrix. The differentiated MC3T3-E1 cells migrated into the interior part of the CCHCs matrix and formed more stable interlinked mature cells. Fluorescent staining of bone cells showed that both cells had spread more broadly and were more flattened with cuboidal structures (Supplementary Figure S2). FITC and DAPI fluorescent stains confirmed that there were no morphological changes observed in the CB3D matrix conditioned medium cultured cells and cells grown on the CCHCs matrix as evidenced by a high number of cells, which also supported the osteogenic differentiation of bone cells cultured on the CCHCs matrix (Figure 3a,b).

### 3.6. Chitosa-Collagen-Based 3D Matrix Downregulates Osteoclast Formation

**Figure 9:**
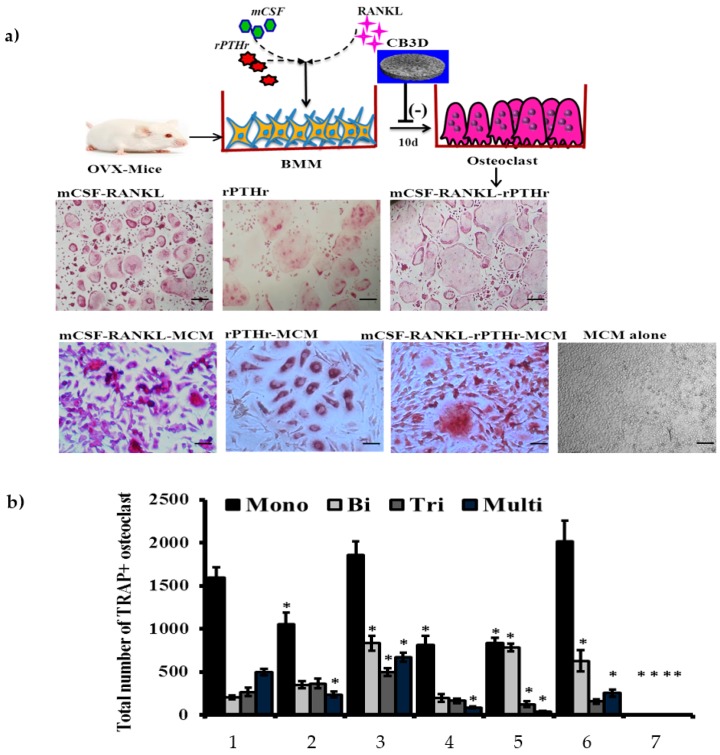
(**a**) Effect of inducers (mCSF, RANKL, and rPTHr11) and CCHCs matrix conditioned medium (MCM) on osteoclast formation from ovariectomized mice. (**a**) Tartrate-resistant acid phosphatase (TRAP) positive osteoclast cells; scale bars: 200 μm. (**b**) Total number (mono-, bi-, tri-, and multi-nucleated) of osteoclasts formed from ovariectomized mice bone marrow macrophages (BMM), 1: mCSF-RANKL, 2: rPTHr11, 3: mCSF-RANKL-rPTHr11, 4: mCSF-RANKL-MCM, 5: rPTHr11-MCM, 6: mCSF-RANKL-rPTHr11-MCM, 7: MCM, * *p* < 0.05 vs. the mCSF-RANKL-treated group. The osteoclast precursor cells were treated with inducers in the presence or absence of MCM. The combination of mCSF-RANKL-rPTHr11 supported mature osteoclast formation. No osteoclasts formed in the MCM alone group. The experiments were done three times with similar results.

To study osteoclastogenesis, osteoclast precursor cells (bone marrow macrophages) isolated from OVX-mouse were cultured with mCSF, RANKL, and rPTHr11 in the presence or absence of 3D matrix conditioned medium (MCM). In general, the osteoclast precursor cells could differentiate into osteoclasts in the presence of the inducers, mCSF-RANKL or rPTHr11 (Figure 9a). The number of TRAP+ BMM-derived osteoclasts was higher with the combination of the inducers mCSF-RANKL-rPTHr11, whereas, the effect of osteoclast induction of these inducers was downregulated by MCM, which was confirmed by the decreased number of TRAP+ BMM-derived osteoclasts in MCM-treated cells (Figure 9b). There were no TRAP+ BMM-derived osteoclast cells observed in cells treated with MCM alone.

### 3.7. Role of the Chitosan-Collagen-Based 3D Matrix on Bone Marrow Mesenchymal Stem Cells, MC3T3-E1, and pOC for Osteoclast Formation

**Figure 10:**
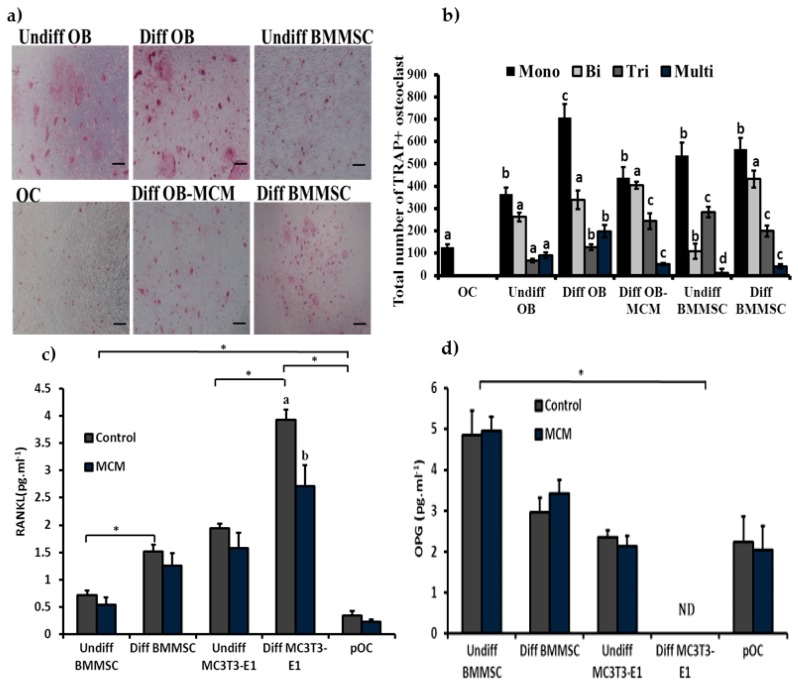
Different stages of osteogenic cells in osteoclast formation. Primary osteocytes and undifferentiated- and differentiated-bone cells (mesenchymal stem cells and osteoblasts) were co-cultured for 10 days with osteoclast precursor cells isolated from the femur and tibia of wild mice bone marrow. (**a**) Images of TRAP-stained osteoclasts; scale bars: 200 μm. (**b**) Total number (mono-, bi-, tri-, and multi-nucleated) of osteoclasts formed after co-culture; different letters indicate statistical significance between treatments. (**c**) The level of RANKL expression produced from different bone cells; different letters indicate statistical significance between control and MCM. (**d**) The level of osteoprotegerin (OPG) expression produced from different bone cells. Undiff OB: undifferentiated osteoblast, Diff OB: differentiated osteoblast, Undiff BMMSC: undifferentiated bone marrow mesenchymal stem cell, Diff BMMSC: differentiated bone marrow mesenchymal stem cell, OC: primary osteocytes, MCM: matrix conditioned medium. * *p* < 0.05 (*n* = 3).

To understand the possible paracrine cues of osteogenic cells in osteoclast formation, pOC and diff or undiff BMMSC and MC3T3-E1 cells were co-cultured with osteoclast precursor cells in the absence of inducers. The effect of the CCHCs matrix conditioned medium on osteoclast formation was investigated. The results showed that the pOC might support BMM-derived osteoclast formation up to mono-nucleus TRAP+ cells, and undiff BMMSC and diff BMMSC (osteoblastic lineage cells) cells support up to three plus TRAP+ nuclei cells, while diff osteoblast cells support up to mature osteoclasts with multinucleated TRAP+ cells (Figure 10a,b). In contrast, the CCHCs 3D matrix (MCM) suppressed the osteoclastogenic effect of diff osteoblast cells by reducing the RANKL secretion of bone cells; however, osteoprotegerin (OPG) levels were not significantly altered between control and MCM-treated cells (Figure 10c,d).

## 4. Discussion

The scaffold compression strength might be expected to influence the differentiation and proliferation of fibroblast [28]. Generally, artificial biopolymers having a stiffness ranging from 3–2000 MPa are recommended for orthopedic application [29,30]. In the present study, the stiffness of the CB3D matrix was 4.8 MPa, suggesting that this matrix has suitable mechanical properties for bone tissue engineering. The compression strength of the 3D matrix improved in the composite CB3D matrices, especially in the CCHCs matrix, due to the combined effect of collagen, chitosan, H, and Cs biomaterials, which may increase the chemical bonding. The porosity of the CB3D matrix was in accordance with the previous report [31]. It had been shown that the coordination interaction of the c-axis of H preferentially-aligned parallel to the longitudinal direction of the collagen fibril, mimicking natural bones with a more dynamic response to the in vivo environment [32]. It has been reported that subcutaneous implantation of a hydroxyapatite-ceramic matrix with 77% porosity induced ectopic bone formation in mice and that the cylindrical synthetic porous hydroxyapatite matrix with 80% porosity healed femoral defects in rats [33,34].

The addition of biomaterials, H and Cs, reduced the pore size of the CB3D matrix from 451 to 250 μm with increased porosity (~88%), which further suggested the appropriateness in tissue engineering, since an ideal 3D matrix should have 80–90% porosity with a pore size of 50–250 μm [35]. The composite CB3D matrix had more decreased water binding and shrinkage than the C matrix. The pore size and intermolecular spaces decreased with the addition of biopolymer, and functional groups of collagen were inter-linked with other biomaterials such as H and Cs by hydrogen bonding and electrostatic interaction, thereby preventing the water molecules from binding into the biopolymer [11,36].

The XRD data confirmed the possible arrangement of H crystals in the collagen fibril and chitosan biomaterials. When collagen is in the form of a 3D matrix, the individual collagen XRD peak may not be visible and appeared as a broad or submerged peak within the chitosan peak [37]. The 2θ peaks of CB3D matrices at 41.97, 43.66, 49.1, and 51.0° occurred due to collagen, and the two broad peaks at 11.3 and 22.8° occurred due to chitosan [38,39].

Collagen and chitosan were covered with amorphous H and Cs, which improved the thermal stability of the CCHCs matrix when compared with other matrices. This high stability of the CCHCs matrix was considered as a characteristic desirable property for further practical applications in tissue engineering. Micro-CT images confirmed the hierarchical 3D structure of CB3D matrices and the 3D matrix structure could resemble the structure of trabecular bone. From the data, it has been established that the formulated CB3D matrix can provide a suitable base similar to native bone tissue for bone cells’ remodeling.

Among the CB3D matrices, CCHCs increased ALP activity in both osteoblastic lineage cells from BMMSC and differentiated MC3T3-E1 cells. The varying differentiation and ALP activity in bone cells depend on the rheological properties such as stiffness and porosity of the CB3D matrix. ALP activity is considered an important factor in determining bone cell differentiation and mineralization [40]. The data also confirmed that the differentiated osteoblasts from MC3T3-E1 cells expressed a high level of cellular ALP compared to the differentiated osteoblastic lineage cells from BMMSC (Figure 3d). Indeed, the individual osteogenic regulatory activities of collagen, chitosan, H, and Cs on bone cells have been previously reported [16,17,41].

In the previous study, BMMSC cultured with mammalian collagen deposited more calcium than the control [42]. In the present study, the CCHCs 3D matrix upregulated osteoblast differentiation from its precursors through elevated cellular ALP and bone minerals. It has been reported that collagen in bone supported calcification of stromal cell matrix [43], as it was found that higher levels of collagen synthesized by differentiated MC3T3-E1 cells might also support high mineral deposition on Day 14, which was further supported through higher protein and gene expression of collagen and osteocalcin in Western blot and RT-PCR. These results may be explained by the fact that the collagen in differentiated MC3T3-E1 cells supported the deposition of minerals. Culturing bone cells on a CB3D matrix increased the osteoblast differentiation transcription factor, Runx2, which might explain the osteogenic stimulatory mechanism of the CB3D matrix. Previous studies confirmed that the collagen may upregulate differentiation of bone cells by activation of Runx2 via the integrin α_2_β_1_-FAK-JNK signaling pathway [21,44]. A recent study showed that collagen polypeptides suppressed mCSF-RANKL-mediated osteoclast formation from its precursor, mBMM [21]. Guillermin et al. [45] reported that porcine collagen hydrolysates containing Asn, Gly, Gln, and Ala suppressed differentiation and maturation of osteoclasts by downregulating transforming growth factor beta (TGF-β). This is supported by the fact that the prepared CB3D matrix suppressed the higher osteoclast activity during an osteoporotic condition in post-menopausal women.

In general, the differentiated osteoblast cells supported osteoclastogenesis to a greater extent than undifferentiated osteoblast and mesenchymal stem cells. These results contradicted the previous report by Nakashima et al. [10], where the osteocytes expressed higher RANKL to support osteoclastogenesis than osteoblasts. The expression of RANKL by bone cells depends on the source and type of bone cells isolated. Moreover, in this study, the expression of RANKL in bone cells may differ based on different stages in osteoblastogenesis.

## 5. Conclusions

The biomaterials hydroxyapatite and chondroitin sulfate enhanced the rheological and functional properties of a CB3D matrix. The topographic structures of a CB3D matrix resembled a trabecular bone. CB3D matrices supported osteoblast differentiation from BMMSC and pre-osteoblast (MC3T3E1) cells and suppressed mCSF-/RANKL- or rPTHr11-induced osteoclast formation from bone marrow macrophages. Not only were osteoblast lineage cells differentiated from BMMSC, the CB3D matrix might also support the differentiation of mature osteoblast cells from pre-osteoblasts. Among the osteoprogenitor cells, differentiated osteoblasts supported osteoclast formation through secreting a major cytokine, RANKL, but this effect was suppressed by the CB3D matrix. Thus, the current findings give several new insights into the material science regarding the possible mechanism of H and Cs arrangement in collagen polypeptides, and the new 3D biomaterials provided excellent features for bone tissue regeneration by mimicking the natural trabecular bone by suppressing the signaling pathways of RANKL-induced osteoclastogenesis in the osteoporotic environment.

## Supplementary Materials

The following are available online at https://www.mdpi.com/2218-273X/9/5/173/s1. Figure S1: The level of ALP in bone cells. ALP level was normalized with corresponding cell numbers at each time point. CC: collagen-chitosan 3D matrix, CCH: collagen-chitosan-hydroxyapatite 3D matrix, CCCs: collagen-chitosan-chondroitin sulfate 3D matrix, and CCHCs: collagen-chitosan-hydroxyapatite-chondroitin sulfate 3D matrix. Figure S2: confocal laser scanning microscope images of bone cells cultured on 3D matrices (scale bars: 75 μm). For confocal laser scanning microscopy, bone cells were grown in a matrix conditioned medium with osteogenic stimulatory medium for 14 days and stained with FITC and DAPI. BMMSC: bone marrow-derived mesenchymal stem cells, MC3T3-E1, pre-osteoblast. CC: chitosan-collagen 3D matrix, CCH: chitosan-collagen-hydroxyapatite 3D matrix, CCCs: chitosan-collagen-chondroitin sulfate 3D matrix, and CCHCs: chitosan-collagen-hydroxyapatite-chondroitin sulfate 3D matrix. Table S1: List of primers and their sequences used in this study.
