# Supplementary material for: Chitosan-Collagen 3D Matrix Mimics Trabecular Bone and Regulates RANKL-Mediated Paracrine Cues of Differentiated Osteoblast and Mesenchymal Stem Cells for Bone Marrow Macrophage-Derived Osteoclastogenesis
Source: Biomolecules. 2019 May 5;9(5):173. doi: 10.3390/biom9050173 (PMC6571924; doi:10.3390/biom9050173)
Supplement: Supplementary file 1 [file biomolecules-09-00173-s001.pdf]

Supplementary Documents

Chitosan-Collagen 3D Matrix Mimics Trabecular Bone and Regulates RANKL-Mediated Paracrine Cues of Differentiated Osteoblast and Mesenchymal Stem Cells for Bone Marrow Macrophages-Derived Osteoclastogenesis

Jeevithan Elango [1,2,*], Kandasamy Saravanakumar [3], Saeed Ur Rahman [4], Yves Henrotin [5], Joe M.Regenstein[6], Wenhui Wu [1, *], and Bin Bao[1]

[1] Department of Marine Bio-Pharmacology, College of Food Science and Technology, Shanghai Ocean University, Shanghai, 201306, China; srijeevithan@gmail.com, whwu@shou.edu.cn, bbao@shou.edu.cn

[2] Bone Biology and Disease Unit, St. Vincent's Institute of Medical Research, Melbourne, Victoria, 3065, Australia.

[3] Department of Medical Biotechnology, College of Biomedical Sciences, Kangwon National University, Chuncheon, Gangwon 24341, Republic of Korea. Saravana732@gmail.com

[4] Interdisciplinary Research Centre in Biomedical Materials (IRCBM), COMSATS University Islamabad, Lahore Campus, Pakistan; saeedbio80@gmail.com

[5] Bone and Cartilage Research Unit, Arthropôle Liège, University of Liège, CHU Sart-Tilman 4000, Belgium; yhenrotin@ulg.ac.be

[6] Department of Food Science, Cornell University, Ithaca, NY 14853-7201, USA ;jmr9@cornell.edu.

* Correspondence: whwu@shou.edu.cn (W.W); srijeevithan@gmail.com (J.E) Tel.: +86 21 61900388, Fax: +86 21 61900364

Supplementary Table S1. List of Primers and its sequence used in this study

S.No	Primers	Sequences
1	GAPDH	5'- AGC TTG TCA TCA ACG GGA AG-3'
		5'- TTT GAT GTT AGT GGG GTC TCG-3'
2	COL I	5'- GCG AAG GCA ACA GTC GCT-3'
		5'- CTT GGT GGT TTT GTA TTC GAT GAC-3'
3	Osteocalcin (OC)	5'- CTC ACA GAT GCC AAG CCC-3'
		5'- CCA AGG TAG CGC CGG AGT CT-3'
4	Alkaline Phosphatase (ALP)	5'- TCC TGA CCA AAA ACC TCA AAG G-3'
		5'- TGC TTC ATG CAG AGC CTG C-3'
5	RUNX2	5'- CCA CCA CTC ACT ACC ACA CG-3'
		5'- TCA GCG TCA ACA CCA TCA TT-3'
6	RANKL	5'- CCT GAG GCC CAG CCA TTT-3'
		5'- CTT GGC CCA GCC TCG AT-3'

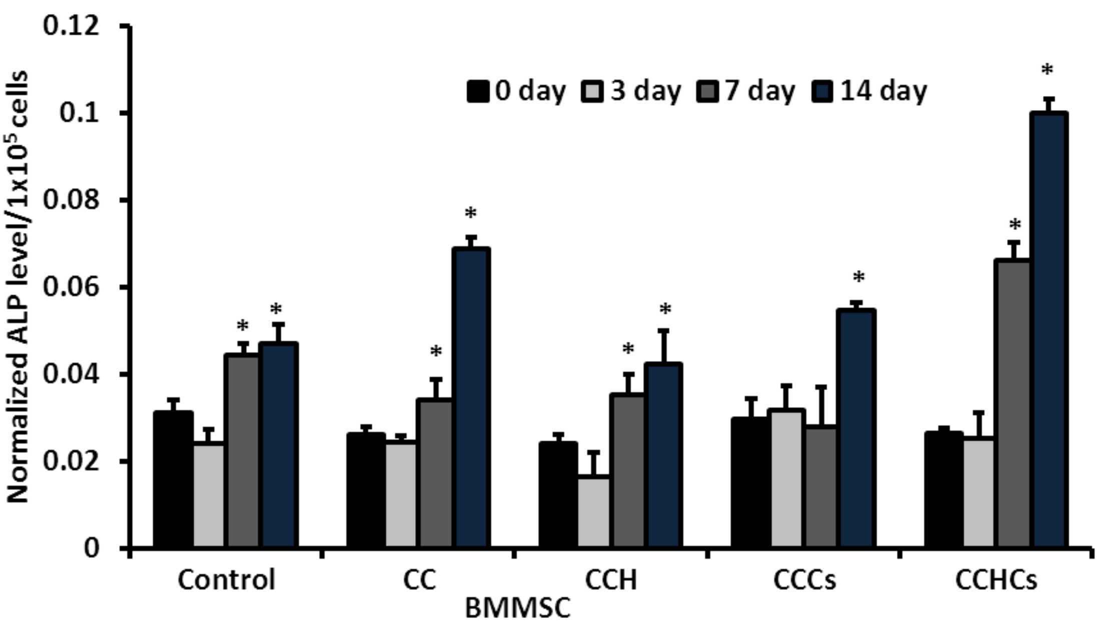

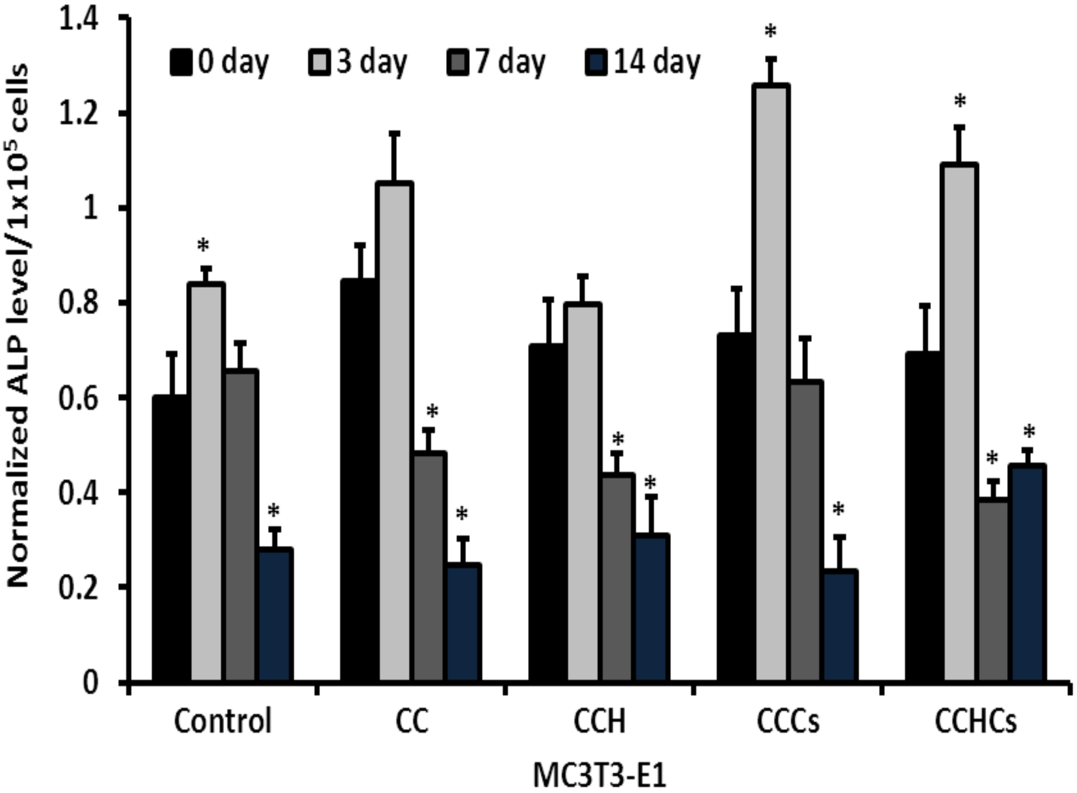

Supplementary Fig S1. The level of ALP in bone cells. ALP level was normalized with corresponding cell numbers at each time point. BMMSC: Bone Marrow-derived Mesenchymal Stem Cell, MC3T3-E1:pre-osteoblast. CC: collagen-chitosan 3D matrix, CCH: collagen-chitosan-hydroxyapatite 3D matrix, CCCs: collagen-chitosan-chondroitin sulphate 3D matrix and CCHCs: collagen-chitosan-hydroxyapatite-chondroitin sulphate 3D matrix.*$p < 0.05$ vs 0 day.

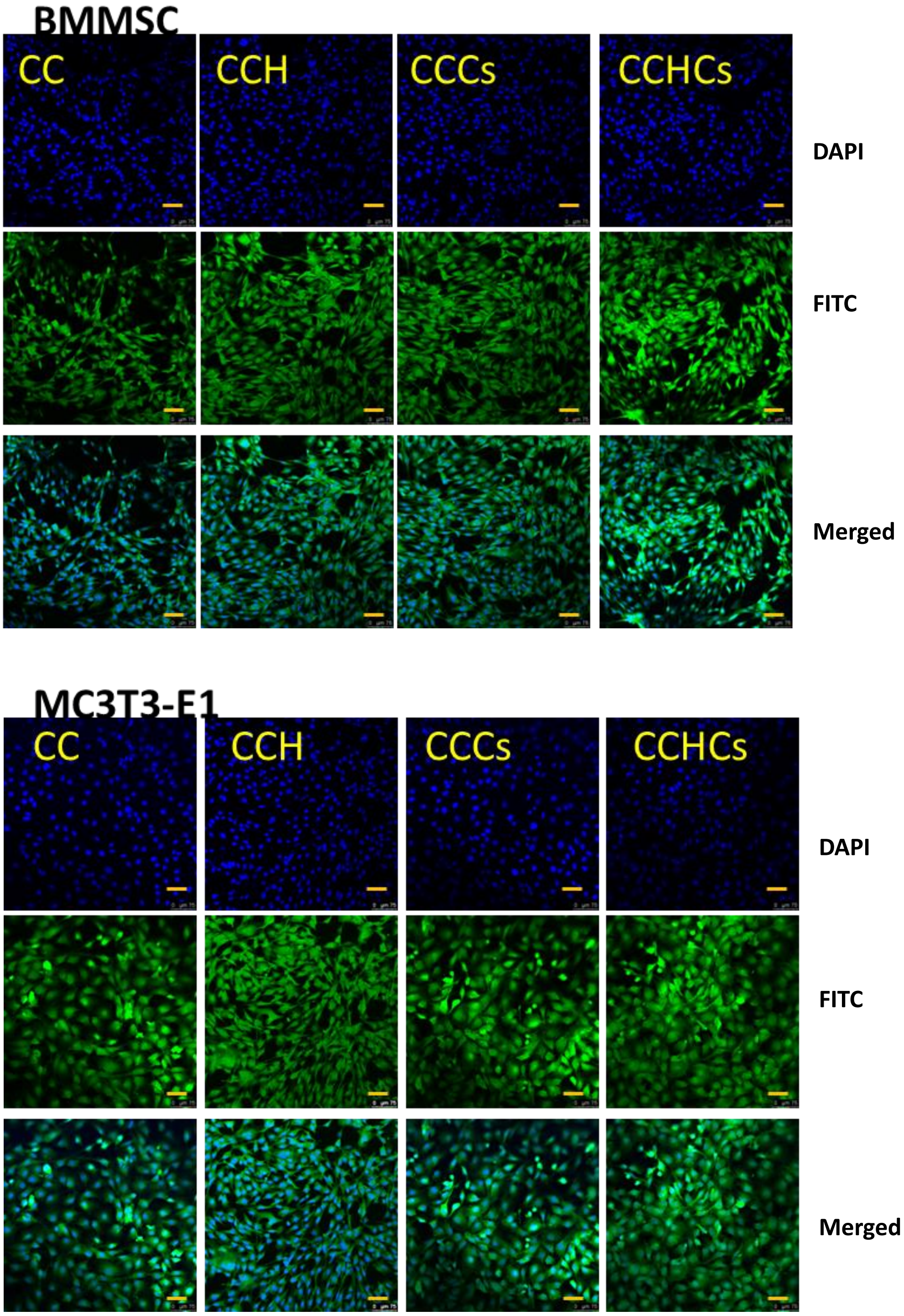

Supplementary Fig S2. Confocal laser scanning microscope (CLSM) images of bone cells cultured on 3D matrix conditioned medium (scale bars: 75 µm). For confocal microscopy, bone cells were grown in a matrix conditioned medium with osteogenic stimulatory medium for 14 days and stained with FITC and DAPI. BMMSC: Bone Marrow-derived Mesenchymal Stem Cell, MC3T3-E1:pre-osteoblast. CC: Collagen-chitosan 3D matrix, CCH: collagen-chitosan-hydroxyapatite 3D matrix, CCCS: collagen-chitosan-chondroitin sulphate 3D matrix and CCHCS: collagen-chitosan-hydroxyapatite-chondroitin sulphate 3D matrix.
